# Phosphate Porous Coatings Enriched with Selected Elements via PEO Treatment on Titanium and Its Alloys: A Review

**DOI:** 10.3390/ma13112468

**Published:** 2020-05-28

**Authors:** Krzysztof Rokosz, Tadeusz Hryniewicz, Łukasz Dudek

**Affiliations:** Faculty of Mechanical Engineering, Koszalin University of Technology, Racławicka 15-17, PL 75-620 Koszalin, Poland; Tadeusz.Hryniewicz@tu.koszalin.pl (T.H.); lukasz.dudek@tu.koszalin.pl (Ł.D.)

**Keywords:** plasma electrolytic oxidation (PEO), micro arc oxidation (MAO), titanium

## Abstract

This paper shows that the subject of porous coatings fabrication by Plasma Electrolytic Oxidation (PEO), known also as Micro Arc Oxidation (MAO), is still current, inter alia because metals and alloys, which can be treated by the PEO method, for example, titanium, niobium, tantalum and their alloys, are increasingly available for sale. On the international market, apart from scientific works/activity developed at universities, scientific research on the PEO coatings is also underway in companies such as Keronite (Great Britain), Magoxid-Coat (Germany), Mofratech (France), Machaon (Russia), as well as CeraFuse, Tagnite, Microplasmic (USA). In addition, it should be noted that the development of the space industry and implantology will force the production of trouble-free micro- and macro-machines with very high durability. Another aspect in favor of this technique is the rate of part treatment, which does not exceed several dozen minutes, and usually only lasts a few minutes. Another advantage is functionalization of fabricated surface through thermal or hydrothermal modification of fabricated coatings, or other methods (Physical vapor deposition (PVD), chemical vapor deposition (CVD), sol-gel), including also reoxidation by PEO treatment in another electrolyte. In the following chapters, coatings obtained both in aqueous solutions and electrolytes based on orthophosphoric acid will be presented; therein, dependent on the PEO treatment and the electrolyte used, they are characterized by different properties associated with their subsequent use. The possibilities for using coatings produced by means of plasma electrolytic oxidation are very wide, beginning from various types of catalysts, gas sensors, to biocompatible and antibacterial coatings, as well as hard wear coatings used in machine parts, among others, used in the aviation and aerospace industries.

## 1. Introduction

Surface modification of metals and alloys may be carried out by electrochemical polishing [1,2,3,4,5,6,7,8,9], magnetoelectropolishing [10,11,12,13,14] and electropolishing with high-current densities [15,16,17], as well as by chemical and electrochemical machining treatment [18,19,20,21,22,23,24,25]. Other ways of modifying the surface are ion implantation [26,27,28,29,30,31], electrophoretic deposition [32], and laser surface treatment [33,34,35,36]. Development of materials engineering and technology in the following years has allowed new challenges to be posed regarding surface engineering, including, but not limited to, fabrication of porous coatings by the Plasma Electrolytic Oxidation (PEO) or Micro Arc Oxidation (MAO) [37] on light metals and alloys, such as aluminum [38,39,40,41,42,43,44] and its alloys [45,46,47,48,49,50,51,52,53,54], magnesium [38,39,55,56,57] and its alloys [58,59,60,61,62,63,64], titanium and its alloys [57,65,66,67,68,69,70,71,72,73,74,75,76,77,78,79,80], zirconium [39,57,81,82], niobium [39,83,84,85,86], tantalum [57,87,88,89,90,91,92,93,94,95,96,97], hafnium [75,98,99], and beryllium [100].

**Figure 1:**
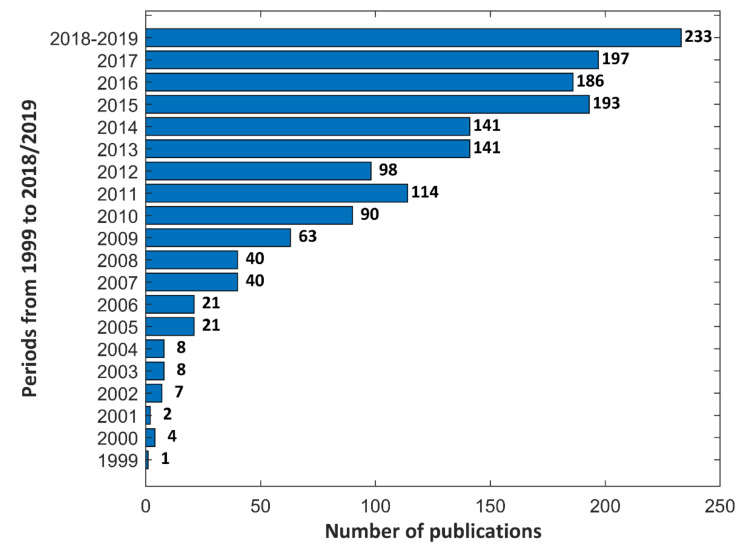
Number of publications based on www.sciencedirect.com in 1999–2019.

In Figure 1, an increase in the number of publications, based on www.sciencedirect.com, in the last twenty years, between 1999–2019 (January), is displayed which proves that the subject of the Plasma Electrolytic Oxidation is still up to date due to the creating new materials and electrolytes. Next, this activity allows to fabricate expected and requested porous coatings which may be used for modification both, machine parts and biomaterials.

The coatings created/fabricated in the processes of plasma electrolytic oxidation (PEO) have an expanded surface stereometry, which can be used both for the production of different types of catalysts [101,102], as well as the biocompatible coatings [103,104]. It is important that in a process that lasts several to tens of minutes, it is possible to produce a coating with the desired porosity and chemical composition. Moreover, further modification of the received surfaces is possible by thermal, hydrothermal treatments, or re-oxidation by PEO in other solution.

## 2. Titanium and Its Oxides

Discovered in 1791 by Williams Gregor, titanium is an element belonging to the group of transition metals, block d of periodic table, it is in the fourth period and fourth group, of the atomic number equaling to 22 and electronic configuration [Ar]3d^2^4s^2^. It has amphoteric properties, and naturally occurs in the earth’s crust in the form of ilmenite (FeTiO_3_) and rutile (TiO_2_) [105]. Due to the fact that titanium is a metal of low density and at the same time of high corrosion resistance, it is increasingly used in various industries. At 1156 K the titanium undergoes phase change from the structure α to β, which is stable to reach a melting point at 1941 K. Thanks to its electron configuration, titanium is able to form solid solutions with most other alloying additions. Titanium and its alloys react with all interstitial elements (e.g., oxygen, nitrogen, hydrogen) in a large temperature range. Solubility in titanium of oxygen equals 12.25% (1112 K), and of nitrogen is 7.6% (1356 K), which in consequence leads to dissolution of the titanium oxide layer in the matrix under the influence of temperature. It should be noted that aluminum and oxygen are the most important stabilizers of α phase in the titanium alloys. In contrast, β phase stabilizers are divided into those that favor formation of binary alloys (α + β) (Mo, W, V, Ta, Nb), as well as those which promote formation of eutectoidal alloys (β → α + γ) (Cu, Mn, Cr, Fe, Ni, Co, Si, H, Pd), and Zr acts as a stabilizer of β phase mainly in alloys of titanium and niobium (Ti-Nb) [106]. As a curiosity, one may notice that in human blood the content of titanium is in the range of 0.03–0.15 mg/kg, in the brain 0.8 mg/kg, in renal cortex 1.3 mg/kg, in the liver 1.3 mg/kg, in the lungs 3.7 mg/kg, and in muscles 0.2 mg/kg [105].

Naturally occurring titanium(IV) oxide was discovered in 1795, while its production began in the twenties of the twentieth century. It is the most widespread substance containing titanium (about 95% of titanium ore is processed into TiO_2_), due to its use, among others, for production of paints, plastics, papers, textiles, coatings with increased corrosion resistance, antibacterial agents, self-cleaning surfaces, food additives, Ultrafiolet (UV) absorbers, cosmetics, and air and/or water purification systems. In addition, application research is underway with TiO_2_, among others, in water remediation, photocatalysis, batteries, supercapacitors, and different sensors. Titanium oxides occur in tetragonal form (anatase, rutile), orthorhombic (brookite), and monoclinic (TiO_2_(B)). Rutile is the most stable form of TiO_2_ at room conditions in macroscopic sizes, while anatase is more stable at nanoscopic sizes. A more compact structure of rutile compared to anatase results in, among others, its higher density and chemical stability. Melting temperature of rutile is 2098 K, while anatase undergoes irreversible transformation into rutile at 773 K. Brookite has similar properties to rutile, in nature it is much less common than anatase and rutile. However, the least widespread form of titanium(IV) oxide of the least density of all listed is TiO_2_(B), for the first time obtained in 1980, which was found naturally occurring in 1991 [107].

Titanium and its alloys, thanks to their properties, have found wide applications, among others, in the aviation industry (fittings, screws, hull frames, brake assemblies, hydraulic hoses, shovels, shafts, afterburner covers, hot air ducts), automotive (valve and suspension assemblies, pulleys, steering gears, connecting rods, drive shafts, springs, crankshafts), architecture (claddings and roofing, concrete reinforcement, renovation of monuments), chemical industry (tanks, pumps, pressure reactors, pipes and pipelines), maritime industry (deep-water hulls, submarines, underwater ball valves, data recording devices, parts of lifeboats, anodes for cathodic protection), and medicine (surgical tools, implants) [18].

## 3. History of the PEO Coatings

The beginnings of Plasma Electrolytic Oxidation date back to 1880, when Sluginov [108] had observed the phenomenon of luminescence on the surface of metals during the galvanic process, which was described by Braun in 1898 [109]. Dufford in his work [110] showed that during electrolysis of metals, such as aluminum, zinc, silver, tantalum, tungsten, magnesium, cerium, antimony, and mercury in selected electrolytes there is a phenomenon of luminescence appearing, which he did not observe in the case of lead, iron, copper, nickel, molybdenum, tin, and platinum. In 1990, Rudnev et al. [111] described a change in thickness of the obtained coatings on aluminum alloys as a function of treatment and proposed a theoretical concept explaining formation of coatings, while a year after the work by Saakiyan et al. [112] appeared, on plasma electrolytic oxidation (PEO) of aluminum in electrolyte containing, among others, hydrogen peroxide. Based on the research, the authors proposed also a two-step mechanism of formation of the oxide layer and showed that the PEO process results in increasing corrosion resistance. In the following years, Yerokhin et al. [113] presented the formation of phases in the ceramic oxide coatings on an aluminum alloy AlMg6.0Mn1.0 during the PEO process in aqueous electrolyte containing Na_6_P_6_O_18_, SiO_2_, and Al_2_O_3_. In their considerations they included two mechanisms of oxides formation: electrochemical oxidation of surface and the oxide synthesis in plasma. It is also important that the thermodynamic calculations they made, both for creating reaction products as well as for heating and cooling during discharges, were divergent from the experimental data by less than 20%. In the next publication [114] they described oxidation of aluminum alloy Al-6.0Zn-2.2Mg-1.7Cu-0.4Mn in aqueous alkaline silicate containing KOH and Na_2_SiO_3_, in which they obtained uniform oxide layers of thickness of 165–190 μm and hardness of 18–23 GPa. In addition to assessing the effectiveness of the process, they used oxide mass as the main parameter. In the next publication [115] they had taken the explanation of the physical and chemical bases of electrolysis in plasma taking into account, inter alia, current-voltage characteristics, electrolytes, analysis of chemical composition and structure, as well as mechanical properties of the obtained coatings. Research was also conducted into the impact of purity of chemical reagents in the PEO treatment on the chemical composition of the obtained coatings [116]. Cleanliness of chemical reagents used to prepare electrolyte has been reported to significantly affect the voltage-time response during plasma electrolytic oxidation of titanium and results in a thin layer formation, enriched with chlorine right on the titanium substrate, which may be one of the reasons for delamination of fabricated coatings [117]. In the following years, numerous scientific studies were conducted on electrolytic oxidation of light metals and their alloys in different electrolytes and combinations of this technique with other ones used for surface modification have been developed; they are, among others, thermal, hydrothermal, and sol-gel treatments.

## 4. PEO Coatings Enriched with Phosphorus

The coatings created in the PEO process at the current density of 60 A/dm^2^ (50 Hz) in the aqueous solutions containing Na_3_PO_4_, for the treatment time from 2 up to 90 min, were characteristic with different thickness and porosity dependent on the electrolyte concentration and PEO oxidation time. It was found that their thickness rises along with the growth of the salt concentration in the electrolyte and with the increase of PEO processing time. Moreover, the diameters of their pores rise with the increase in time of treatment, which is most visible in case of comparison of the surfaces obtained after 2 and 90 min of processing. It should be noted that such surfaces may possess the photocatalytic properties against the methylene blue during UV radiation [118]. The sample surfaces obtained in the same electrolyte, also having photocatalytic properties, were created at the process voltage from 300 V to 500 V (600 Hz, 8%) and the treatment time equaling 5 min. It was noted that the change of voltage in the PEO process significantly affected the porosity of the obtained surfaces. With increasing voltage, increasing diameters of pores was noted from a fraction of nanometer up to over 1.5 nm [119]. On the other hand, adding NaOH to the electrolyte containing Na_3_PO_4_, using 340 V voltage forcing of frequency from 1000 Hz to 2000 Hz (10%) resulted in decreasing the maximum diameters of pores from 1184 down to 743 μm in the fabricated coatings with simultaneous increase of their roughness parameters [120,121].

## 5. PEO Coatings Enriched with Phosphorus and Calcium

The usage of electrolyte containing Ca(CH_3_COO)_2_·H_2_O, NaH_2_PO_4_·H_2_O, NaOH and complexing compounds, such as Sodium Gluconate (GA) or Triethanolamine (TEA), or (Ethylenedinitrilo)tetraacetic acid disodium salt (EDTA-2Na), or Ethylenediamine tetramethylene alendronate (EDTMPS) in different concentrations in the PEO process (300–350 V, 50 Hz, 30 min) resulted in the creation of porous coatings containing TiO_2_ (rutile, anatase, brookite) for the coatings obtained in the electrolytes containing GA, TEA, EDTMPS, and additionally CaTiO_3_ for the layers formulated in solutions with the addition of EDTA-2Na. It was also noted that the lowest ratio Ca/P (1.3) was obtained for the coating formed in the electrolyte containing EDTMPS, and the highest (1.5) for the electrolyte solution with GA. During the studies, the least porosity (20.4%) was registered for the coating obtained in the solution with EDTA-2Na, and the highest (30.7%) with TEA. The highest corrosion resistance was revealed with the sample oxidized in the solution with EDTMPS. It was also found that an additional 28-day exposition of the samples in the solution of Simulated Body Fluid (SBF) resulted in the formation and the presence of titanium(IV) oxides and hydroxyapatite (HA) in all coatings [122].

The use of aqueous electrolyte of Ca(CH_3_COO)_2_·H_2_O with Na_2_HPO_4_·12H_2_O and with NaOH to the PEO treatment of titanium, with the use of rectangular alternating voltage AC 450 V (200–1000 Hz, 20%) at the process times from 10 to 30 min, allowed to obtain the coatings of a developed stereometric structure. It was noted that along with the rise of frequency, there was increase in micropore sizes from about 4 up to 20 μm as well as the ratio Ca/P from about 0.5 to 14.5. For the frequency of 600 Hz, the ratio Ca/P was 1.65 at the pore size of 8 µm, that could be considered as the result closest to a biocompatible coating. One could also observe that increasing the PEO process and decreasing the frequency results in the rise of coating thickness. It is important that the fabricated coatings contained rutile and anatase, however, the anatase was transformed into rutile at high temperatures. This is why at lower frequencies in the coating there were more anatase, whereas at high frequencies the anatase was vanishing almost completely with only rutile and CaTiO_3_ being left [123,124,125]. It has also been shown that preliminary preparation of samples by sandblasting affects the PEO coating created on titanium. For this purpose, the PEO treatment (450 V, 600 Hz, 20%, 10–30 min) was carried out on titanium samples after sandblasting process and without it, in the same electrolyte as related/described in [123] and porous surfaces were obtained. Then the obtained samples were placed in SBF solution for a few days to check their ability to induce hydroxyapatite.

It was found that oxidation of the coatings after sandblasting was definitely harder than in the case of those ones without the preliminary treatment. For all the obtained PEO coatings the rise in treatment time resulted in the increase in porosity from 5.9% (10 min) to 19.2% (30 min) for the sandblasted coatings and from 8.7% for (10 min) to 25.8% (30 min) for the non-sandblasted ones. Moreover, in both cases the resulting coatings were characterized by their high adhesion. For the treatment times from 10 to 20 min, the average thicknesses of coatings on the sandblasted samples were greater than those arising on non-sandblasted titanium, whereas for the times from 25 to 30 min a reversed situation was noticed. The thinnest coating (of about 15 µm) was obtained for the PEO process time of 10 min on the sandblasted and non-sandblasted surfaces, whereas the thickest one (of about 45 µm) on the sandblasted samples. It was also found that the contents of calcium and phosphorus were the biggest in the middle part of the coating, independent on the titanium sample was sandblasted or not. On the other hand, the coatings fabricated on the sandblasted surfaces were characteristic with a higher concentration of calcium and phosphorus in the outer layer, which can be explained by the creation of hydroxyapatite [123].

In the electrolyte on the basis of Ca(H_2_PO_4_)_2_·H_2_O with the addition of (NaPO_3_)_6_, NaOH and EDTA-2Na the PEO process was carried out under anodic voltage of 300 V and cathodic one of 30 V (600 Hz, 20%) at the treatment time of 5 min, for coating fabrication. In the coatings created with the composition of anatase and rutile, the Ca/P ratio ranged from 0.16 to 1.08. After the additional twenty-four-hour exposition to 7 mol/dm^3^ NaOH of temperature 363 K and three-hour heating at the temperature of 973 K, apart from titanium oxides, also Ca_2_P_2_O_7_ and CaTiO_3_ were registered. On the other hand, a seven-day exposition of the samples in SBF solution resulted in detection of A-TiO_2_, CaTiO_3_, and HA phases [126]. Further on, the use of the same phase of solution with Ca(H_2_PO_4_)_2_·H_2_O, Na_2_PO_4_·12H_2_O, and EDTA with the addition of C_6_H_5_Na_3_O_7_ at the stabilization of current density during the PEO process on the level of 0.6 A/dm^2^ (600 Hz, 20%) and assuming the maximum voltage ranging from 300 to 450 V, led to creation of porous coatings. The coatings after an additional hydrothermal treatment (473 K, 5 h) in aqueous solution consisting of Ca(H_2_PO_4_)_2_·H_2_O, Na_2_PO_4_·12H_2_O, and NaOH (pH in the range 12–13) revealed the titanium(IV) oxides and hydroxyapatite they contained [127].

Another solution used to fabricate the porous coatings under PEO process (450 V, 600 Hz, 8%, 5 min) was an aqueous solution of Ca(CH_3_COO)_2_·H_2_O, with Ca(H_2_PO_4_)_2_·H_2_O, and NaOH. The fabricated coatings were additionally exposed for 48 h in aqueous solutions of NaOH (5–15 mol/dm^3^) of temperature 313 K and then by placing them in the SBF solution for the period from 7 to 28 days. It has been noted that an increasing concentration of NaOH of the first solution results in increasing both the number and depth of cracks on the PEO coating, which may lead to eventual delamination on the surface, as well as an increased ability to induce the apatite on the PEO surfaces in SBF solution. It has also been shown that the longer the time of exposition of samples in the SBF solution, the greater the amount of apatite in the obtained PEO coating [128].

The addition of C_3_H_7_CaO_6_P to the aqueous solution of Ca(CH_3_COO)_2_·H_2_O at sinusoidal alternating voltage of 800 Vpp and frequency 50 Hz for the treatment times from 10 to 90 min allowed to obtain amorphous coatings of thicknesses from 9 to 15 µm, consisting of hydroxyapatite, anatase (A-TiO_2_), rutile (R-TiO_2_), and CaTiO_3_, TiP_2_O_7_, for which the ratio Ca/P was in the range from 1.43 to 1.93. Additionally, it was noticed that the hydrothermal treatment of coatings in aqueous solution of pH equaling 11 for 2 h at the temperature of 473 K and pressure of 1.3–1.8 MPa resulted in growth of that ratio to 2.26 [129]. Oxidized PEO titanium for 7.5 min in the same aqueous electrolyte at the voltage of 450 V (300 Hz, 9.6–10%), and after hydrothermal treatment in the autoclave in 100 cm^3^ aqueous solution of pH 11.0–11.5 for 10 h at the temperature of 463 K, was covered with a hard and highly wear resistant coating, containing titanium(IV) oxides and hydroxyapatite, with the ratio Ca/P being in the range from 1.4 to 1.7. Moreover, the obtained coating was characteristic with a better osteointegration than the material before the applied treatments [130].

The use of voltage 500 V (600 Hz, 12%) to PEO treatment in the same electrolyte resulted in getting coatings which in the next step were placed in the Hanks solution of the temperature 311 K for 14 days. The coatings, both before and after exposition to the Hanks solution, consisted of titanium(IV) oxides (anatase and rutile), hydroxyapatite, and α-TCP. Additionally, it was found that the resulting layers were characterized with high resistance against corrosion [131]. On the other hand, the use of the same electrolyte in the PEO process (190–600 V, 660 Hz, 10%, 3 min) led to creation/fabrication of porous coatings of thicknesses from about 1 μm (230 V) up to 15 μm (600 V).

In case of voltages lower than 230 V, it was impossible to measure thickness of coatings based on the SEM analysis of metallographic microsections. It has been noted that the coatings formed at low voltages contained anatase with the amount rising with the increase of voltage. At the voltage of 270 V, rutile appeared in the amount rising with further increase of PEO voltage, resulting at the same time in decreasing the amount of anatase. It was found that the size of craters grew along with the PEO voltage used. The analysis of biocompatibility of the obtained samples showed that the best parameters possessed the layers fabricated at the voltage of 300 V used [103]. Addition of C_3_H_7_Na_2_O_6_P instead of C_3_H_7_CaO_6_P to the aqueous solution of Ca(CH_3_COO)_2_ to the PEO process under voltage control (200 V, 350 V, 500 V) for the treatment times of 3, 6, and 10 min resulted in the formation of porous coatings on titanium of the pore size in the range of 70–650 nm, containing the crystal phases A-TiO_2_ (anatase), CaTiO_3_, Ca_3_(PO_4_)_2_, α-Tricalcium phosphate (α-TCP), and hydroxyapatite (HA).

It has also been noted that the ratio Ca/P increased along with the growth of PEO voltage from 1.23 (200 V) to 2.10 (500 V). It was found that the sample fabricated at the voltage of 350 V for 3 min was characterized with the highest amount of phase of hydroxyapatite and revealed the closest to it the ratio Ca/P equaling 1.6. In addition, it was noticed that the volume of pores rises along with the growth of salt concentration in electrolyte, as well as with increasing time of the coating growth. Moreover, it was noted that the pore diameters grow along with the increase of time of arcing. Dependent on the growth time and electrolyte concentration, the pore sizes were in the range of 0.303–1.770 μm and amounting to 10.7%–17.3% of the studied surface. A layered model was proposed in which in the transition layer is located CaTiO_3_, responsible for adhesion of coating to the base, whereas α-TCP and HAp constitute the outer layer of coatings [132,133,134].

A porous coating may be fabricated in a five-minute PEP process under a stabilized current density on the level of 8 A/dm^2^, in the electrolyte containing additive of Na_5_P_3_O_10_. Then it was covered with a tantalum-based paste and heated in oven at the temperature of 873 K for 1 h. The obtained porous coating of about 15 μm thick and the ratio Ca/P within the range of 0.9 to 2.3 revealed a clear division into layers. In the upper layer of the coating there is mainly tantalum(V) oxide, and below, inside is the layer dominated by titanium(IV) oxides and calcium phosphate. The next one, a transition layer, joins the coating with titanium and mainly contains TiO [40]. By contrast, adding to that electrolyte of Na_2_SiO_3_·9H_2_O, and the use of anodic voltage of 460 V and cathodic of 160 V, at the frequency 800 Hz and the treatment time of 20 min, resulted in obtaining porous coatings consisting of anatase (A-TiO_2_), rutile (R-TiO_2_), and the compounds of calcium and phosphorus of the ratio Ca/P equaling 1.69 [135].

On the other hand, the use of aqueous solution on the basis of Ca(CH_3_COO)_2_·H_2_O with Ca(H_2_PO_4_)_2_·H_2_O and EDTA-2Na to the PEO process and applied voltages from the ranges of 250 to 550 V for the time of 15 min, along with additional exposition to a 6 M NaOH solution through 10–20 s caused the formation of coating consisting of anatase (A-TiO_2_) and rutile (R-TiO_2_). It was noticed that the growth of PEO voltage results in increasing the ratio of rutile to anatase and the amount of CaTiO_3_ in the coating as well as porosity of the obtained coatings [136].

Adding NaOH to this electrolyte for the PEO process (300 V, 600 Hz, 8%, 5 min) resulted in the formation of porous coating, in which the morphology changed after a twenty-four hour exposition to aqueous solution of NaOH (5 mol/dm^3^) at temperature 313 K, as well as after heating for 1 h at the temperatures of 973–1073 K. After all those treatments were done, the porous coating contained TiO_2_ (rutile, anatase), CaTiO_3_, and CaTi_21_O_38_. One should mention that an additional exposition of the coatings in SBF solution after fourteen days caused the remaining apatite and rutile only, and after twenty-eight days–apatite only [137,138,139].

The use of salt KH_2_PO_4_ instead of NaOH for the PEO treatment (400 V, 700 Hz, 40%) caused the formation of coatings containing titanium(IV) oxides and hydroxyapatite. It was also noticed that the content of calcium and phosphorus in the coating grew along with the increase of concentration of salt in the electrolyte, and the content of hydroxyapatite increased with the growth of ratio Ca/P. It was noticed that the thickness of the coating rises with the growth of electrolyte concentration to an asymptotic value equaling of about 25.2 μm, and further increasing of the electrolyte concentration did not affect its thickness. It was also stated that the growth of electrolyte concentration results in improving its biocompatibility [140].

The use of aqueous solution of Ca(CH_3_COO)_2_ with C_6_H_18_O_24_P_6_, and EDTA-2Na and KOH to PEO processes, with a stabilization of current density in the range of 2 to 8 A/dm^2^ for the treatment times of 2 to 6 min, allowed to achieve porous coatings of the ratio Ca/P being in the range from 0.1 to 1.0, consisting of crystal phases of TiO_2_ (anatase and rutile) and CaTiO_3_, as well as amorphous ones of Ti_2_O_3_, and/or CaO, and/or CaCO_3_, and/or Ca_3_(PO_4_)_2_, and/or CaHPO_4_. It has also been shown that increase of the content of calcium acetate in electrolyte, as well as increase in current density of PEO process results in the growth of calcium content in fabricated coatings. It was also noticed that it was the PEO process time which had the least effect on the content of calcium in the PEO coatings [141].

The use of aqueous solution of Ca(H_2_PO_2_)_2_ for the PEO process, with the applied constant voltages in the range from 100 to 400 V, led to create the porous coatings containing TiO_2_, CaTiO_3_, for which the ratios Ca/P from 0.2 to 0.5 were calculated. It should be mentioned, however, that the amount of calcium registered in the coatings have not exceeded the value of 4 wt%, at 8 wt% of phosphorus. It was also noticed that the surface porosity was increasing with the increase in PEO voltage, whereas the pore diameters after the treatment at 200 V were ranging from 0.7 to 3.1 μm, and for oxidation at 400 V they were in the range from 0.6 to 4.4 μm. It was found that the highest amount of calcium and phosphorus was built-in into the coating after PEO treatment at 300 V and that amount was decreasing slightly when reducing the PEO voltage [142].

Addition of 2 mol/dm^3^ H_3_PO_4_ to that electrolyte, for the five-minute PEO process at the voltages of 80 V and 150 V resulted in obtaining porous coatings of thicknesses from 319 up to 510 nm for oxidation at 80 V, and from 8 to 27.5 μm for the treatment at 150 V [143]. Due to the fact that the coating obtained at 150 V had the thickness ranging from several to several dozens of micrometers, a lower voltage of the PEO process of 140 V was used [144], for which the thickness of the obtained coating was in the range from 11.6 to 15.4 μm, however with the surface of some lesser porosity gained.

It has been noticed that the ions of calcium and phosphorus were included into the surface oxide layers. The coatings obtained that way were relatively thick and amorphous. An additional thermal treatment did not lead to creation a crystal phase, whereas the alkaline treatment resulted in formation of a gel-like coating. The highest resistance to corrosion was observed on the samples oxidized at 80 V. However, the studies in vitro in SBF solution demonstrated that the HA growth occurred only on samples fabricated at 140 V.

It has been also noted that the use of electrolyte containing Ca(H_2_PO_2_)_2_ with Ca_3_(PO_4_)_2_, or CaSiO_3_, or SiO_2_ in a five-minute PEO process at a constant current density on the level of 15 A/dm^2^, at the limiting voltages from 150 to 450 V, results in obtaining porous coatings of thicknesses ranging from a few (at 150 V) to several (at 450 V) micrometers of the ratios Ca/P from 0.04, for the coatings obtained in electrolyte containing 150 g/dm^3^ CaSiO_3_ and the PEO process voltage of 150 V, to the highest ones (1.58) for the aqueous solution with the addition of 100 g/dm^3^ CaSiO_3_ at the voltage 450 V [145,146].

## 6. PEO Coatings Enriched with Phosphorus, Calcium and Silver

Additional enrichment of porous Ca-P coatings with silver affects their anti-bacterial properties, that may be achieved, for instance, by the addition of silver compounds or silver nanoparticles to the electrolyte. Using the PEO process (250–450 V, 1000 Hz, 60%, 1–3 min), an aqueous electrolyte containing C_3_H_7_NaO_6_P·5H_2_O, (CH_3_COO)_2_Ca·H_2_O, and AgNO_3_ or CH_3_COOAg, as well as H_2_PtCl_6_, led to fabrication of biocompatible and antibacterial porous coatings.

It has also been noted that at DC voltages in the range from 250 to 350 V for the treatment time equaling to 3 min, two phases (rutile and anatase) were obtained in the coating. Increasing the voltage to 420 V at the same oxidation time resulted in the change of phase composition into rutile, β-Ca_2_P_2_O_7_, α-Ca_3_(PO_4_)_2_, and hydroxyapatite. It was found that increasing the voltage affected the appearance of phase α-Ca_3_(PO_4_)_2_ [147]. On the other hand, the use of anodic (400 V) and cathodic (80 V) voltages (500 Hz, 60%) and using electrolyte on the basis of aqueous solution of (CH_3_COO)_2_Ca·H_2_O with Na_2_HPO_4_ and AgNO_3_ resulted in the formation of coatings with TiO_2_ in the inner layer and hydroxyapatite with silver in the outer layer.

In contrast to the usually obtained surface morphology in the PEO process, in that case the micropores were difficult to identify. The reason for this was the HA appearance on the obtained surface in the form of granules (for the AgNO_3_ content on the level of 0.1 g/dm^3^), and needle-like structures (for the AgNO_3_ content on the level of 0.4 g/ dm^3^). It has also been noted that increasing the content of AgNO_3_ increases the amount of crystal hydroxyapatite in the coating and causes silver particles to precipitate in its outer layer. It was found that for the AgNO_3_ concentration equaling 0.1 g/dm^3^ the PEO coatings formed are characteristic with a sufficient antibacterial activity without a risk to cytotoxicity [148].

The use of stabilization for the current density on the level of 65 A/dm^2^ (800 Hz, 30%) in electrolytes with the addition of AgNO_3_ to carry out a one-step three-minute PEO process resulted in creating the coatings characteristic with antibacterial properties. It was found that along with increasing the AgNO_3_ concentration (5–16 g/dm^3^) the nanopore diameters were decreasing down to about 40 to 15 nm, and the thickness of the pore walls increased from about 10 to 36 nm. It has also been noted that adhesion of the fabricated coatings increases with the growth of current density; at this current density it appears to be higher than at lower ones. Then it was shown that silver is built into the crystalline TiO_2_ in the form of AgO crystals of diameters from 10 to 30 nm.

It was also concluded that a high content of silver in the coating is antibacterial but it is also toxic to the body, mainly because of the fact that Ag^+^ ions are very easy to oxidize into Ag^2+^, that leads to the cytotoxicity [149]. Addition of silver nanoparticles of dimensions 37 ± 6 nm to the aqueous solution of Ca(CH_3_COO)_2_·H_2_O with C_3_H_7_CaO_6_P in the five-minute PEO process under a constant current density of 20 A/dm^2^ (50 Hz) caused the creation of porous and biocompatible (Ca/P = 2.2) coating of antibacterial properties (Ag/Ca = 0.004), in which titanium(IV) oxides (anatase, rutile) and silver were present in the structure. It was also found that nanoparticles were homogeneously adsorbed on the porous surface, and their presence affected the morphology of emerging coatings. All the obtained layers had pores with dimensions lesser or equal to 5 µm. It was also observed that the content of silver nanoparticles on the surface increased as the amount of nanoparticles increased in the electrolyte. It is also important that the coating was characteristic with a good antibacterial activity with low cytotoxicity at the same time in relation to bone tissues [150,151,152].

However, in a five-minute PEO process (380 V, 500 Hz, 60%) in the electrolyte consisting of C_4_H_6_O_4_Ca, Na_2_HPO_4_, and CH_3_COOAg, a multilayer coating was obtained, composed of outer biocompatible hydroxyapatite and CaTiO_3_, and next two layers in order (porous and non-porous) with TiO_2_ (anatase and rutile). It was also noted that the ratio Ca/P for coatings containing and non-containing silver was in the range from 1.54 to 1.57. Here, the coating with silver 4.6 wt% was characteristic with the best antibacterial properties against *E. coli* and *S. aureus* [153]. At the same time, it was found that coatings containing of about 2 wt% of silver did not show cytotoxicity to osteoblasts that is crucial for biocompatible coatings [154].

## 7. PEO Coatings Enriched with Phosphorus, Calcium and Magnesium, as well as Zinc and Copper

Zinc-enriched coatings exhibit antibacterial properties [44,155], however, zinc is better tolerated by living organisms than copper and silver are. Some exemplary electrolyte for the manufacture of such coatings in the four-minute PEO process (16.5 A/dm^2^, 800 Hz, 10%) is an aqueous solution of Ca(CH_3_COO)_2_·H_2_O, and C_3_H_7_Na_2_O_6_P with Zn(CH_3_COO)_2_·2H_2_O. Porous coatings of investigated antibacterial activity against *S. aureus* and *E. coli,* which have been obtained during that process, contain anatase and rutile, and are enriched with zinc (4.6–9.3 wt%). It was calculated that the ratio Ca/P equaling 0.5 was lower in the coating not containing the zinc (Ca/P = 0.9). Most likely, the cause of this fact is a partial substitution of ions Ca^2+^ by ions Zn^2+^ [141,156].

The use of aqueous solution containing Ca(CH_3_COO)_2_·H_2_O with C_3_H_7_Na_2_O_6_P, Zn(CH_3_COO)_2_·2H_2_O, and silver nanoparticle (6 g/dm^3^) of diameters lower than 20 nm under PEO process (390 V, 100 Hz, 26%) at the treatment times from 0.5 to 4 min led to fabricate porous coatings of antibacterial properties (*S. aureus)*. It was found that along with the increase of treatment time, the amount of zinc and silver detected increased. The porous coating, created during a two-minute PEO process, consists of amorphous phase of titanium oxides, anatase, rutile, ZnO, and Zn_2_TiO_4_. It has also been noted that the ratio Ca/P for all surfaces is approximately equal to 1.4, while Zn/Ag is over 20, that indicated better incorporation of Zn^2+^ ions than silver nanoparticles. In case of the coating fabricated under the two-minute PEO process, which was found to be biocompatible with the best antibacterial properties amongst the investigated, it was characteristic with a layered structure, in which most zinc (30.6–31 wt%), as well as silver (1 wt%) have been registered for the middle part of the coating. The next outer layer was porous, in which 29.7–29.8 wt% of zinc and 1.3–1.6 wt% of silver were registered. The smallest amount of these elements was detected in a transient layer (7.1 wt% Zn; 0.5 wt% Ag) [157].

In case of using voltage stabilization in the PEO process (450V, 100 Hz, 26%) in electrolyte containing Ca(CH_3_COO)_2_·H_2_O, C_3_H_7_Na_2_O_6_P·5H_2_O and Cu(CH_3_COO)_2_, there were obtained porous coatings enriched with copper (0.67–1.93 wt%) containing crystal phases of anatase and rutile, as well as an amorphous phase, most likely enriched in Ca_3_(PO_4_)_2_ and/or CaHPO_4_, and the Cu^2+^ ions. It was shown that the addition of copper(II) acetate did not affect the morphology of obtained surfaces, however it did have an effect on antibacterial properties of fabricated layers. In addition, it was noted that the amount of copper equal to 1.93 wt% in the coating, in addition to its antibacterial properties, is characterized by cytotoxicity [157].

It should be pointed out that authors have contribution in development of PEO processes with usage of concentrated (85%) orthophosphoric acid instead of water as an electrolyte with additives such as Ca(NO_3_)_2_, Mg(NO_3_)_2_, Zn(NO_3_)_2_ and Cu(NO_3_)_2_ under both DC [158,159] and AC [160] conditions. In case of use of electrolyte that contains all four nitrates under DC conditions, it was found out that additional metal ions from electrolyte occurs in obtained porous surfaces amorphic phase while Ti_2_P_2_O_7_ occurs in crystalline phase. It was also noted that metal/phosphorus ratio increased from 0.172 at.% up to 0.224 at.% for coatings obtained under 500 and 650 V, respectively [158]. In addition, similar results were obtained for samples fabricated in electrolyte containing only concentrated phosphoric acid with addition of magnesium nitrate(V) obtained under AC-PEO process (200 V_pp_, 50 Hz). This resulted in obtaining coating that was characterized by an Mg/P ratio equaling to 0.176, which is a positive effect due to the its good biocompatibility [160].

## 8. PEO Coatings Enriched with Phosphorus, Calcium and Strontium or Lanthanum

The strontium enriched coating may be fabricated in the electrolyte containing Ca(CH_3_COO)_2_·H_2_O, NaH_2_PO_4_·H_2_O, and Sr(OH)_2_·8H_2_O, by undergoing the samples to a one-minute PEO treatment at a fixed voltage of 350 V. It has been noticed that at a rising content of strontium ions in the electrolyte, the ratio Ca/Ti is decreasing from 0.082 down to 0.070, while the ratio Sr/Ti increases from 0 to 0.021, and the ratio Ca/P is in the range from 1.2 to 1.3. It was also found that with a small amount of strontium in the coating, the cell cultures developed much faster, which confirmed the supposition that Sr element improves biocompatibility of the PEO coatings [161]. The coatings containing calcium, phosphorus, and strontium, changed morphology after a thermal treatment. An increase in temperature caused an increase in the amount of anatase and rutile, while at the temperature 1472 K, Ca_3_(PO_4_)_2_ appeared additionally. Thermal treatment of rutile at such a high temperature caused large cracks between the layer that arises in the PEO process and titanium or titanium alloy substrate. The reason was a mismatch in thermal expansion. The studies prove that, thanks to such treatments, both the mechanical properties as well as adhesion of coatings can be improved, and choosing the right temperature will significantly increase the adhesion of coatings, which is highly desirable in implantology [161]. Change of Sr(OH)_2_·8H_2_O onto Sr(CH_3_COO)_2_·0.5H_2_O at the same processing conditions also resulted in fabrication of porous coating enriched with strontium (0.1–1.6 at%), for which the ratio Ca/P was on the average of 0.65, and being half of the magnitude of the coatings without the addition of strontium. However, it has been shown that the content of strontium improves bone ingrowth and that way accelerates implant osseointegration [162].

Use of an aqueous solution containing Ca(CH_3_COO)_2_, C_3_H_7_Na_2_O_6_P, and Sr(CH_3_COO)_2_ in the PEO process (400–450 V, 100 Hz, 26%, 5–10 min) allowed to obtain coatings on a porous material of pores 50–150 μm, most likely containing Ca_3_(PO_4_)_2_ and SrTiO_3_. Based on the results obtained, it was determined that the size of pores and amount of oxygen, calcium, phosphorus, and strontium was the highest in the outer layer, in which more anatase and rutile was detected than in deeper layers. Higher voltage used in the PEO process was found to have a great influence on the thickness of the outer coating; for the voltage 450 V it was 460 times greater than that at the voltage of 400 V. A high thickness is important, because the thicker the outer layer, the more likely an interaction between osteoblasts and substrate [163].

Further research on Sr-Hap coatings manufactured in the PEO process is of importance due to a great potential in dental and orthopedic applications. Compared to conventional HAp coatings, they significantly increase integration of bone implant, which has been confirmed by biomechanical tests in vivo [164]. It has also been shown that the PEO method is better than the APS method (plasma spraying) for medical applications, because of the risk/threat of delaminating. Plasma sprayed coatings may undergo delamination as a result of emerging stresses, which leads to a rapid dissolution of plasma applied HAp coating and causes mechanical degradation, which destroys the adhesion of the coating, which in turn can cause inflammation. In this connection, they are the PEO coatings which have a better durability, which gives them the possibility of long-term clinical use [165].

Another electrolyte that can be used to manufacture coatings with strontium during the PEO process at a constant current density of 5 A/dm^2^ at time from 10 to 40 min is aqueous solution of Ca(CH_3_COO)_2_·H_2_O with Na_5_P_3_O_10_ and Sr(CH_3_COO)_2_. It has been noted that after the PEO process and a three-hour thermal treatment at 873 K, the coatings obtained in the electrolytes not containing strontium acetate had the thickness from 13 to 119 μm and had in their structure mainly СaTi_4_(PO_4_)_6_, NaTi_2_(PO_4_)_3_, Ca_2_P_2_O_7_, and Ca_4_O(PO_4_)_2_, while those fabricated in the solution containing compound with strontium ions were characteristic with the thicknesses from 13 to 154 μm and contained additional phases of SrTi_4_(PO_4_)_6_, Sr_7_Ti(PO_4_)_6_, Sr_2_P_2_O_7_, and Sr(PO_3_)_2_. There were also slight differences in porosity of the surface layer noted [166].

In contrast, electrolyte based on Ca(CH_3_COO)_2_·H_2_O and NH_4_H_2_PO_4_ with the addition of La(NO_3_)_3_ in a ten-minute PEO process at the voltage of 400 V allowed to fabricate porous coatings enriched with lanthanum. The addition of salt of this element caused the created layers to have a more homogeneous morphology in comparison with the coatings without the addition of La(NO_3_)_3_, which created more favorable conditions for bone growth and a formation of apatite. In addition, it was observed that the presence of La(NO_3_)_3_ in electrolyte reduces coefficient friction of the fabricated coating of 0.4, for the same wear conditions. It was also noted that the coating enriched with lanthanum is a better substrate to produce hydroxyapatite than its counterpart obtained in electrolyte without the addition of La(NO_3_)_3_ [167].

## 9. PEO Coatings Enriched with Phosphorus, Iron or Nickel

Fabrication of coatings enriched with phosphorus and iron on titanium substrate in a thirty-minute PEO process in the aqueous solution of Na_3_PO_4_∙12H_2_O, NaOH, and NaF was possible by using stabilization of AC current density in the range from 10 to 30 A/dm^2^ (125 Hz, 75%) [168]. It has been noted that the porosity of coatings and the size of pores decrease with increasing applied current density. Furthermore, the use of forty-minute PEO processing at the voltage of 400 V with variable frequencies from 100 to 1600 Hz in the same electrolyte resulted in obtaining porous coatings of different thickness and surface morphology. It has been noticed that by increasing frequency from 100 Hz to 1600 Hz, a decrease in coatings thickness from 19.2 to 8.6 μm was obtained. It was also noted that the sizes of micropores of the oxide coating (TiO_2_ with Fe^3+^) were increasing as the voltage frequency of the PEO process increased. At power source frequencies from 800 to 1600 Hz the micropores are uniformly distributed on the entire surface of the fabricated coating.

In addition, it has been tested that the obtained porous coatings at the voltage of 400 V and the frequency of 800 Hz after impregnation in aqueous solution of 0.15 mol/dm^3^ Fe(NO_3_)_3_ for 36 h were characteristic with good photocatalytic properties in regard to methylene blue at irradiation by a light source imitating sunlight [168]. A similar effect of incorporation of iron cations to an oxide matrix/mold has been observed on the surface of PEO coatings obtained in an aqueous electrolyte containing Na_3_PO_4_∙12H_2_O and FeSO_4_ using current control (15 A/dm^2^) in a two-minute PEO process. The use of aqueous solutions of Na_3_SiO_3_ and Na_3_PO_4_ enriched with K_3_[Fe(CN)_6_] in a ten-minute PEO process (8 A/dm^2^) allowed to obtain porous coatings (TiO_2_ with Fe_2_O_3_) characterized by better photocatalytic properties in the process of phenol degradation [169].

The addition of NiO to aqueous solution of Na_3_PO_4_∙12H_2_O in the form of a suspension allows for obtaining crystalline structures containing both anatase, rutile, and NiO, with the proportion of each phase dependent on the PEO process parameters. It has been noted that the higher voltages of the process generate discharges of higher energy, resulting in the formation of increasingly large pores, and at 500 V some local pitting/meltings on the fabricated surface are observed. Structures of TiO_2_ enriched with NiO are characteristic with better photocatalytic properties in regard to the TiO_2_ structures which is due to the formation of semi-conductor pairs of n-TiO_2_/p-NiO. This allows limiting the recombinations of pairs electron hole/electron due to the existence of an internal electrostatic field being a barrier to recombinations.

The performed studies have shown that the coatings obtained at the voltage of 300 V in a five-minute PEO process are characterized by better photocatalytic activity in photodegradation reaction of 4-chlorophenol under UV irradiation than the coatings fabricated in electrolyte containing only dodecahydrate sodium phosphate(V) [170].

## 10. PEO Coatings Enriched with Phosphorus and Copper, Silver or Zinc

To fabricate porous coatings enriched with phosphorus and copper, an aqueous electrolyte containing NaOH, NaH_2_PO_4_, and copper nanoparticles may be used in a five-minute PEO process with a current stabilization on the level of 20 A/dm^2^. They included crystalline phases of anatase and titanium and were enriched both with ions of Cu^+^, as well as Cu^2+^. Addition of nanoparticles did not affect the morphology of fabricated coatings. It was further demonstrated that the obtained coatings with copper (2–2.5 wt%) have antibacterial properties [79].

On the other hand, authors use with success phosphoric-based electrolytes to fabricate coatings containing copper under Direct Current Plasma Electrolytic Oxidation (DC-PEO) condition [171,172,173,174,175,176,177,178,179,180,181,182], which was implemented through usage of electrolytes contain dissolved copper(II) nitrate in concentrated (85 wt.%) orthophosphoric acid. It should be pointed out that this type of electrolyte allows to prepare porous Cu-enriched coatings not only on titanium, but also on its alloys, such as Ti-Nb-Zr-Sn [183] or nitinol [184], as well as on Ti6Al4V [185,186], TNZ [186]. Authors in their studies for fabrication PEO coatings have used industrial transformer equipped with six diodes of Greatz Bridge [187] or standard DC power supply with high voltage output. Performed studies of coatings obtained in electrolyte containing H_3_PO_4_ with Cu(NO_3_)_2_·3H_2_O within voltage range from 450 V to 650 V, reveals its copper enrichment (Cu/P = 0.24–2.59 at%), which is also proved by Cu/P ratio (0.009–0.082) [188]. It was also proven that obtained porous coatings enriched with copper exhibit antifungal and antibacterial properties [189].

In contrast, porous coatings enriched with phosphorus and silver, or with phosphorus and zinc, due to the lack of calcium, cannot be considered as biocompatible, however they exhibit photocatalytic properties both using radiation in the UV range as well as visible light. It has been shown that for coatings containing phosphorus and zinc, the voltages of the ranges from 100 to 200 V do not cause the formation of regular porosity on fabricated coatings, which in turn is visible in the range from 300 to 500 V.

The possibility of producing anatase-rutile layers, enriched with crystalline compounds of silver (Ag_3_PO_4_, and Ag_2_O) exists, for instance, in a three-minute PEO process in aqueous solutions of Na_3_PO_4_∙12H_2_O, NH_4_OH, and Ag_2_CO_3_, at the voltages from 300 to 500 V. It has been observed that increase in the amount of silver ions in electrolyte causes the increase in both the porosity as well as the development of stereometry of fabricated surfaces. In addition, it has been shown that the increase in voltage from 300 to 500 V in the PEO process may result in deterioration of photocatalytic properties of the obtained coatings, which is associated with a reduction in the ratio of amount of anatase/rutile [190].

There is also the possibility of enriching porous coatings with phosphorus and zinc by using the PEO process with a voltage stabilization (300–360 V) in the electrolyte containing Na_3_PO_4_∙12H_2_O, H_3_PW_12_O_40_, and ZnO. Porosity has been reported to increase as the treatment time and the processing voltage increase. It has also been noted that most zinc (Zn^2+^) was built into the structure of coatings fabricated under a five-minute PEO process at 360 V. X-Ray Diffraction (XRD) studies pointed to the presence both anatase, as well as rutile in a crystalline phase. It has also been shown that the use of voltage of 360 V and time of 30 min in the PEO process in this electrolyte allows to obtain the best photocatalytic properties in the process of degradation of methylene blue [191].

## 11. PEO Coatings Enriched with Phosphorus and Vanadium or Tungsten

To improve the photocatalytic properties of coatings fabricated on titanium by the PEO method a modification of the used electrolytes has been proposed, usually being aqueous solutions of Na_3_PO_4_∙12H_2_O, by the addition of NaVO_3_. Use of this aqueous solution in the PEO process at DC voltages in the range from 250 to 550 V allowed to improve the photocatalytic properties of degradation of methylene blue during irradiation of the coating by UV radiation. In addition, it was noticed that increase of the PEO process voltage results in increase of the share of vanadium(V) oxide (V_2_O_5_) in the crystalline structure, next to anatase and rutile. Insertion of vanadium compounds into the surface of PEO coating made it possible to perform a photocatalytic process by irradiation in the UV range and visible light as well [192,193,194]. Further work on the applications of higher mentioned electrolytes led to improve the photocatalytic properties of the obtained coatings doped by V_2_O_5_ by the use of PEO process with pulsation in the range from 250 to 500 Hz and maximum voltage of 450 V [195]. In addition, it has been proved experimentally that the use just only aqueous solution of NaVO_3_ allows for obtaining crystalline structures in the PEO coating, containing anatase, rutile, and V_2_O_5_ at voltages in the range of 300–500 V.

The obtained surfaces, in contrast to those arising with the use of aqueous solution containing only Na_3_PO_4_∙12H_2_O, stood out by their characteristic coniferous surface structure. Alike the coatings obtained in the electrolyte being the salt mixture of Na_3_PO_4_∙12H_2_O and NaVO_3_, they are characteristic with the ability to catalyze the reaction of decomposition of methylene blue under the influence of radiation of UV as well as visible light, with the highest photocatalytic ability characteristic for coatings obtained at the DC voltage of 500 V in the aqueous solution of NaVO_3_ [193].

The use of PEO process in the electrolyte containing Na_3_PO_4_∙12H_2_O and Na_2_WO_4_ also resulted in the formation of coatings of photocatalytic properties. Use of this electrolyte for the PEO process allowed to fabricate crystalline structures, containing next to anatase and rutile, also tungsten(VI) oxide (WO_3_).

As a result of observations, it was proved that the use of 2 g/dm^3^ Na_2_WO_4_ at the DC voltage of 550 V in the PEO process allowed to obtain developed surfaces characteristic with good photocatalytic properties in the process of degradation of methylene blue [196,197]. In contrast, the use of acidic aqueous solution of Na_2_WO_4_ with H_3_PO_4_ in the PEO process with bipolar pulse power supply (700 Hz, 450 V, −50 V, 0.3 V) for 40 min allowed to obtain surfaces of amorphous features. An additional calcination process at the temperature of 973 K and time 60 min allowed to obtain in the PEO coating of crystalline structures of anatase and WO_3_, characteristics with a more open porosity. Prepared in this way, surfaces after the PEO process and calcinations revealed the ability to cause photodegradation of rhodamine during UV irradiation [198].

## 12. PEO Coatings Enriched with Phosphorus and Europium or Terb

The opportunity has also been demonstrated to manufacture porous coatings enriched with phosphorus and lanthanides, which were characterized by photocatalytic properties. To fabricate, in the PEO process, the layers enriched with europium and terbium, the aqueous solutions of Na_3_PO_4_∙12H_2_O with Eu_2_O_3_ or Tb_4_O_7_ were used. It has been noticed that as the treatment time was increased from 1 to 10 min in the PEO process of fabrication coating with europium, its porosity and the amount of this element in the coating also increased (Eu/P = 0.01–0.08). It has been shown that conducting/running the one-minute PEO process with the use of stabilization of current density on the level of 15 A/dm^2^ in the solution containing europium(III) oxide results in formation of the surface layer containing anatase in the crystalline phase, which is doped by Eu^3+^ ions, resulting in an increase in catalytic photoactivity in the process of degradation of methyl orange with the use of a light source simulating solar radiation [199]. In contrast, the surfaces additionally enriched with terbium may be obtained in this process at the PEO processing times from 1 min (Tb/P = 0.02) to 10 min (Tb/P = 0.09). It has been noted that into the coating built mainly with anatase, Tb^3+^, as well as Tb^4+^, ions could be introduced.

It has been shown that the content of Tb^3+^ increases both with the increase in the PEO processing time, as well as increasing the content of Tb_4_O_7_ in the electrolyte. In addition, it was noted that for longer times of the process, porosity of the fabricated coatings increases. It has also been shown that optimal for obtaining anatase coatings, enriched with Tb^3+^ ions of photocatalytic properties, is a one-minute PEO process, in the aqueous solution containing 10 g/dm^3^ Na_3_PO_4_∙12H_2_O and 0.5 g/dm^3^ Tb_4_O_7_ [200].

## 13. Summary

In this paper, the porous oxide coatings enriched with phosphorus, with the addition of other elements in the view of functionalizing them, were presented. It has been shown that when using the PEO process, one may fabricate porous coatings consisting mainly of titanium(IV) oxides (A-TiO_2_ and R-TiO_2_), as well as other compounds, dependent on the electrolytes used or other additional treatments performed: thermal, hydrothermal (pH = 12–13), or exposition to alkaline solutions on the basis of (NaOH, KOH), or to SBF. Among others, hydroxyapatite was obtained, e.g., Ca_3_(PO_4_)_2_, CaTiO_3_, TiP_2_O_7_, Ca_2_P_2_O_7_, Ca_4_O(PO_4_)_2_, СaTi_4_(PO_4_)_6_, NaTi_2_(PO_4_)_3_, as well as SrTiO_3_, SrTi_4_(PO_4_)_6_, Sr_7_Ti(PO_4_)_6_, Sr_2_P_2_O_7_, Sr(PO_3_)_2_, and Ca_5_P_8_, Mg_3_P_2_. However, the created pores in most cases are blurred and oval, which is a characteristic feature of surfaces obtained in aqueous electrolytes. Analysis of the presented literature with a review referred to the oxide coatings containing phosphorus, with or without additional elements, allowed to conclude that an increase in the PEO process voltage increases the thickness of fabricated coatings and the size of their pores, as well as the amount of introduced elements coming from the solution. However, the increase in voltage frequency of the PEO process results in a reduced coating thickness and size of their largest pores, while increasing concentration of the elements from electrolyte. In addition, it was established that increasing the time of PEO treatment results in increasing the content of elements derived from electrolyte in the coating, increase in its thickness and porosity, as well as size of the pores.
